# Usefulness of Infrared Thermal Imaging Camera for Screening of Postoperative Surgical Site Infection after the Nuss Procedure

**DOI:** 10.1155/2013/946156

**Published:** 2013-06-25

**Authors:** Kenya Fujita, Masahiko Noguchi, Shunsuke Yuzuriha, Daisuke Yanagisawa, Kiyoshi Matsuo

**Affiliations:** ^1^Department of Plastic and Reconstructive Surgery, Shinshu University School of Medicine, 3-1-1 Asahi, Matsumoto 390-8621, Japan; ^2^Department of Plastic and Reconstructive Surgery, Nagano Children's Hospital, Toyoshina 3100, Azumino 399-8288, Japan

## Abstract

*Introduction and Objective*. The Nuss procedure is widely used in the treatment of pectus excavatum worldwide. Postoperative pectus bar infection is one of the most serious complications associated with this procedure. Therefore, early detection of signs of implant infection is very important. However, this is difficult, and effective methods have yet to be established. *Methods*. We use a handheld infrared thermal imaging camera to screen patients for postoperative infection following the Nuss procedure. Here, we report a 28-year-old man with recurrent postoperative (Ravitch procedure) pectus excavatum. *Results*. Infrared thermography camera clearly indicated slight cellulitis in the right chest. *Conclusion*. Our technique may assist in preventing postoperative bar infection and removal caused by severe bar infection. Furthermore, this camera is potentially suitable for many situations in infection monitoring following subcutaneous implant surgery.

## 1. Introduction

The Nuss procedure is a well-established method for minimally invasive repair of pectus excavatum, which is both safe and shows good cosmetic results [1]. However, there have been reports of severe intraoperative and postoperative complications, among which postoperative pectus bar infection is one of the most serious complications. We use a handheld infrared thermal imaging camera (FLIR B60; FLIR Systems Inc., Wilsonville, OR, USA) to screen for postoperative infections in patients following the Nuss procedure.

## 2. Case Presentation

**Figure 1:**
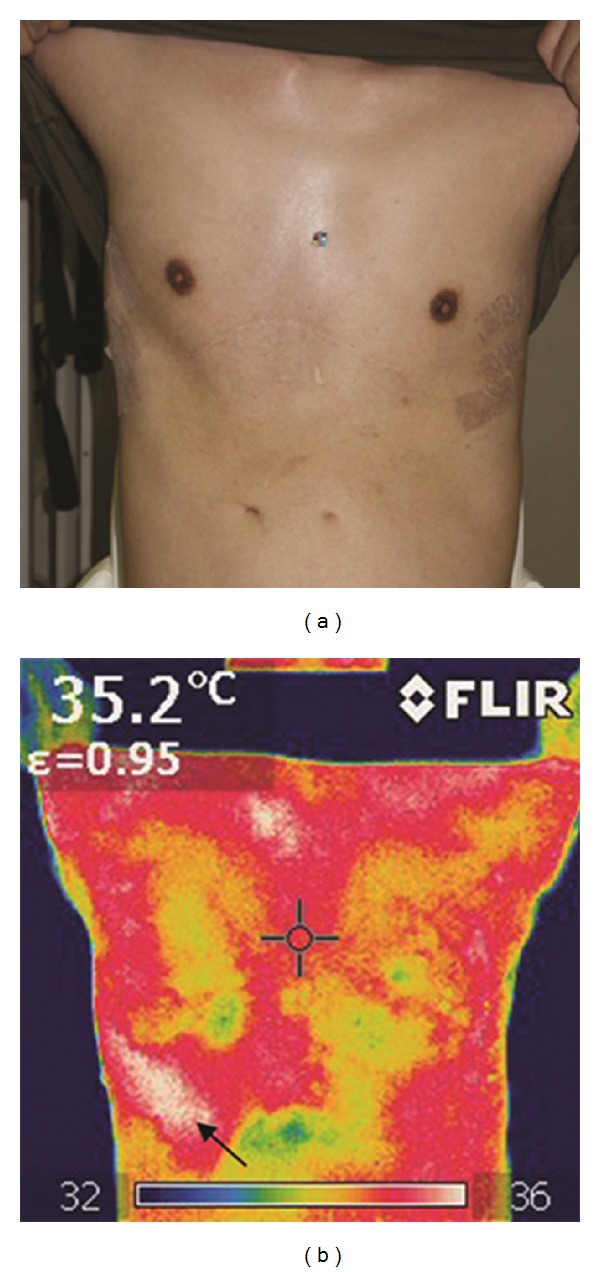
The figure shows clinical (a) and thermographic (b) aspects after the Nuss procedure. A hot spot was detected in the right lower chest wall ((b), black arrow). The resolution of the thermal imaging camera was 180×180 pixels, and the thermal sensitivity was less than 0.08°C. Note the complete absence of clinical signs of inflammation in the normal photograph.

A 28-year-old man with recurrent pectus excavatum following the Ravitch procedure was treated with the Nuss procedure. Implantation of three bars was required to correct his chest deformity. At 5 weeks postoperatively, he complained of slight sluggishness. His serum C-reactive protein level was simultaneously elevated to 1.5 mg/L from 0.1 mg/L (3 weeks after operation), and the rate of neutrophilic leukocytes had increased from 54% to 78%. However, clinical infectious signs and symptoms were mostly absent, and we could not determine whether these observations were due to infection at the surgical site or another site, for example, upper respiratory inflammation (e.g., common cold). However, infrared thermal imaging clearly indicated a hot spot in the operated anterolateral chest wall (Figures 1(a) and 1(b)). The patient immediately received intravenous antibiotic therapy (meropenem) with hospitalization for 1 week, followed by oral administration of minocycline. His symptoms improved, and surgical debridement was not required. 

## 3. Discussion

The Nuss procedure is a well-established method for minimally invasive repair of pectus excavatum, which is both safe and shows good cosmetic results [1]. However, there have been reports of some severe complications in both the intraoperative and postoperative periods, among which postoperative pectus bar infection is one of the most serious complications. Infections after the Nuss procedure were categorized as bar infections, cellulitis, and stitch abscesses [2]. Cellulitis can be managed by conservative treatment (antibiotics and surgical debridement), whereas bar infections require bar removal. When a patient presents with signs and symptoms of bar infection (fever, increasing pain along the bar, erythema along the bar, and drainage from one or more of the incision sites), hospital admission is recommended [2]. Therefore, the early detection of surgical site infection and early administration of antibiotics are very important for good clinical results [2, 3]. In our case, infrared thermal imaging clearly indicated a hot spot in the operated anterolateral chest wall, and the patient was treated appropriately in the early stage of infection.

The use of infrared thermography was reported previously for detection of infectious symptoms in total knee replacement implant infection [4]. This method is also useful to aid in detection of postoperative infection in the Nuss procedure. Furthermore, this camera may be suitable for infection monitoring following subcutaneous implant surgery, for example, mammary implants, reconstruction titanium plates, tissue expanders, pacemakers, defibrillator implants, and so forth.
